# Characteristics of taste alterations in people receiving taxane-based chemotherapy and their association with appetite, weight, and quality of life

**DOI:** 10.1007/s00520-021-06066-3

**Published:** 2021-02-18

**Authors:** Mikiko Kaizu, Hiroko Komatsu, Hideko Yamauchi, Teruo Yamauchi, Masahiko Sumitani, Ardith Z Doorenbos

**Affiliations:** 1 Keio University Graduate School of Health Management Course for Nursing, 35 Shinanomachi, Shinjyuku-ku, Tokyo, 160-8582 Japan; 2 Japanese Red Cross Kyushu International College of Nursing, 1-1 Asty Munakata-City, Fukuoka, 811-4157 Japan; 3 Department of Breast Surgical Oncology, St. Luke’s International Hospital, 9-1 Akashi-cho, Chuo-ku, Tokyo, 104-8560 Japan; 4 Division of Medical Oncology, St. Luke’s International Hospital, 9-1 Akashi-cho, Chuo-ku, Tokyo, 104-8560 Japan; 5 Department of Pain and Palliative Medicine, The University of Tokyo Hospital, 7-3-1 Hongou, Bunkyo-ku, Tokyo, 113-8655 Japan; 6 Department of Biobehavioral Health Science, College of Nursing, University of Illinois at Chicago, Chicago, IL USA; 7 Palliative Care, University of Illinois Cancer Center, 845 S. Damen Ave, Chicago, IL 60612 USA

**Keywords:** Taste alterations, Taste disorders, Chemotherapy, Taxane, Quality of life

## Abstract

**Purpose:**

There is limited evidence on the effect of chemotherapy-associated taste alteration. This study aimed to evaluate taste alteration characteristics in patients receiving taxane-based chemotherapy and investigate the association of taste alterations with appetite, weight, quality of life (QOL), and adverse events.

**Methods:**

This cross-sectional study evaluated 100 patients receiving paclitaxel, docetaxel, or nab-paclitaxel as monotherapy or combination therapy. Taste alterations were evaluated using taste recognition thresholds and severity and symptom scales. Taste recognition thresholds, symptoms, appetite, weight, and adverse events were compared between patients with and without taste alterations, and logistic regression analysis was performed to identify risk factors.

**Results:**

Of the 100 patients, 59% reported taste alterations. We found significantly elevated taste recognition thresholds (hypogeusia) for sweet, sour, and bitter tastes in the taste alteration group receiving nab-paclitaxel (*p* = 0.022, 0.020, and 0.039, respectively). The taste alteration group reported general taste alterations, decline in basic taste, and decreased appetite. Neither weight nor QOL was associated with taste alterations. Docetaxel therapy, previous chemotherapy, dry mouth, and peripheral neuropathy were significantly associated with taste alterations.

**Conclusions:**

Almost 60% of patients receiving taxane-based regimens, especially docetaxel, reported taste alterations. Taste alteration affected the patient’s appetite but did not affect the weight or QOL. Docetaxel therapy, previous chemotherapy, dry mouth, and peripheral neuropathy were independent risk factors for taste alterations.

## Introduction

Taste alteration (TA) is one of the most concerning adverse events associated with chemotherapy [1–3]. When taste is lost or altered, foods or drinks are no longer pleasant and appealing [4]. Accordingly, TAs can cause loss of appetite [5–8], decreased dietary [5, 6, 9] or caloric intake, weight loss [5, 10, 11], and even malnutrition [5, 12]. As eating plays an important role in sociocultural and emotional aspects, TAs can also lead to reduced interest and pleasure in social interactions [4, 7, 13].

Taste sensation is based on five basic qualities: sweet, bitter, salty, sour, and umami. There are approximately 10,000 taste buds around the tongue and laryngopharynx that act as peripheral taste receptors, with each taste bud containing 50–150 taste receptor cells [14]. When food comes in contact with taste receptor cells, taste sensation is transmitted to the brain via three cranial nerves: facial (VII), glossopharyngeal (IX), and vagus (X) [15]. Higher age [16] and smoking [17] are generally considered to be factors that are associated with TA.

The mechanism by which chemotherapeutic agents cause TAs is not entirely known. The most generally accepted hypothesis is that these agents cause cytotoxic damage to these rapidly dividing taste receptor cells [18, 19]. A previous study has shown that fluorouracil, taxane, platinum, and anthracycline agents are associated with a high prevalence of TAs [20]. The prevalence of TAs associated with taxane-based chemotherapies ranges from 75 to 93% [18, 21]. Because taxane agents are used to treat several solid tumor cancers, many patients treated with taxane-based chemotherapies are assumed to experience TAs. Based on previous research, evidence on the effect of taxane-based chemotherapies on TAs is limited because targeted regimens were highly heterogeneous. Additionally, despite the importance of assessing both the actual state of taste function and the various taste symptoms, only few studies have used validated objective and subjective methods [22].

This study aimed to (1) evaluate the characteristics of TAs in patients receiving taxane-based chemotherapies, using objective and subjective methods; (2) investigate the association of TAs with appetite, body weight, quality of life (QOL), and adverse events; and (3) identify the factors that affect TAs.

## Materials and methods

### Study design and participants

This cross-sectional study was conducted at two outpatient chemotherapy units of a university hospital and a general hospital in Tokyo. The inclusion criteria for participants were as follows: (1) age ≥20 years; (2) completion of at least two cycles of taxane-based chemotherapies, including paclitaxel (PTX), docetaxel (DTX), or nab-paclitaxel (nab-PTX) as monotherapy or combination therapy; and (3) fluency in spoken and written Japanese. The exclusion criteria were as follows: (1) presence of brain metastasis, impaired glucose tolerance (hemoglobin A1c ≥6.5), Sjogren’s syndrome, or thyroid dysfunction requiring treatment with levothyroxine sodium; (2) previous treatment history of total gastrectomy or radiotherapy to the head and neck region; (3) taking zinc tablets or anticholinergic agents; or (4) inability to participate due to physical or mental condition at the time of recruitment. This study was approved by the Institutional Review Board of The University of Tokyo (approval no. 11736), St. Luke’s International Hospital (approval no. 17-R116), and Keio University Graduate School of Health Management (approval no. 2017-18).

### Procedures

Participants were recruited between December 2017 and November 2018. The study was introduced by physicians to potential participants. If a potential participant showed interest, a researcher explained the study in detail and obtained written informed consent. For the sake of convenience, data were collected on the day of chemotherapy administration of participants, that is, 7 days after the PTX administration, 7 or 14 days after the nab-PTX administration, and 21 days after the DTX administration. Objective taste evaluations were performed by a researcher before administration of antiemetic or anticancer agents. After that, participants filled in demographic information and self-reported variables on a questionnaire.

### Measurements

#### Taste evaluations

TAs were objectively evaluated using taste recognition thresholds (TRTs) via a taste disc kit (Sanwa Kagaku Kenkyusho, Nagoya, Japan). A filter paper disc immersed with one of five concentrations of sweet (3, 25, 100, 200, 800 mg/ml sucrose), salty (3, 12.5, 50, 100, 200 mg/ml NaCl), sour (0.2, 2, 20, 40, 80 mg/ml tartaric acid), and bitter tastants (0.01, 0.2, 1, 5, 40 mg/ml quinine hydrochloride) was placed on the patient’s tongue, and the minimum perceived concentration was scored from 1 (lowest) to 5 (highest); incorrect responses even at the highest concentration were scored 6 points [23]. We measured at two points on the tongue and defined their mean score as TRTs. A score of <3.5 was considered “normal,” and ≥3.5 indicated “dysgeusia.”

The severity of TAs in the past 7 days (recall period) was evaluated using the National Cancer Institute’s Patient-Reported Outcomes version of the Common Terminology Criteria for Adverse Events (PRO-CTCAE) on a scale of 0–4 (none/mild/moderate/severe/very severe) [24], which is validated in Japanese [25]. We identified participants who reported “none” as without TAs and those who reported “mild” to “very severe” as with TAs.

Subjective TAs and unpleasant symptoms in the past 7 days were evaluated using the Chemotherapy-induced Taste Alteration Scale (CiTAS) [26]. The CiTAS comprises 18 items under four dimensions: decline in basic taste (5 items), general TAs (4 items), phantogeusia and parageusia (3 items), and discomfort (6 items). Each item was scored on a Likert scale of 1 (“taste normally” or “no”) to 5 (“unable to taste at all” or “very”), and the mean score of each dimension was calculated. A higher score indicated hypogeusia tendency or stronger symptoms.

The TA duration between the chemotherapy administration intervals was evaluated using self-report data from the participants.

#### Appetite, weight change, and quality of life

Appetite over the past 7 days was evaluated using the Japanese version of the PRO-CTCAE [25]. The weight difference of participants between the first day of taxane-based chemotherapy and the day of data collection was calculated using medical records.

The patient’s QOL in the past 7 days was evaluated using the validated Japanese version of the Functional Assessment of Cancer Therapy-General (FACT-G) [27, 28]. The FACT-G comprises 27 items under four dimensions: physical (7 items), social/family (7 items), emotional (6 items), and functional well-being (7 items). Each item was scored on a Likert scale of 0 (“not at all”) to 4 (“very well”); the mean score of each dimension was calculated. A higher score indicated a better QOL.

#### Psychological distress

Psychological distress over the last 30 days was evaluated using the Japanese version of the K6, which consists of 6 items [29, 30]. Each item was scored on a Likert scale of 0 (“not at all”) to 4 (“always”). A total score of ≥5 was interpreted as a psychological stress response, and ≥9 as a mood/anxiety disorder.

#### Adverse events

Nausea, mouth or throat sores, dry mouth, and numbness or tingling in the hands or feet within the past 7 days were also evaluated using the Japanese version of the PRO-CTCAE [25]. In patients receiving DTX therapy, we evaluated the following variables to assess the symptom status during 7 days after DTX administration: TA symptoms (CiTAS), TA severity, appetite, and adverse events (PRO-CTCAE). Since DTX is administered every 21 days with a longer interval than PTX and nab-PTX, we analyzed data obtained during 7 days after DTX administration when most adverse effects manifest.

#### Demographic and clinical information

The following demographic information was collected using a questionnaire: age, sex, living conditions, occupational status, smoking habits, and cooking responsibilities. The following clinical information was obtained from medical records: disease stage; previous chemotherapy (“yes” if treated with other chemotherapy regimen(s) for 2 months before the start of the taxane-based chemotherapies); body surface area; number of treatments; and cumulative dose of PTX, DTX, or nab-PTX.

### Statistical analysis

For patient characteristics, continuous variables are expressed as means and standard deviations (SDs) or as medians and interquartile ranges according to distribution, whereas categorical variables are expressed as frequencies and proportions. To compare demographic and clinical variables, objective and subjective taste evaluations, appetite, weight change, QOL, K6 scores, and adverse events between participants with and without TAs, we used the Student’s *t*-test or the Mann-Whitney *U*-test for continuous variables and the *χ*^2^ test or Fisher’s exact test for categorical variables, as appropriate. The association of objective taste evaluations and weight change, QOL, K6 scores, and adverse events between participants with and without TAs, and the association between TA characteristics and appetite were analyzed with Spearman’s rank correlation coefficient. The association of TAs with demographic variables, clinical variables, and adverse events was analyzed using a logistic regression model. The selection criteria for explanatory variables for the remaining model with univariate logistic regression analysis were set at *p* < 0.10. All possible explanatory variables were analyzed using multiple logistic regression analysis with backward stepwise elimination. All statistical analyses were performed using JMP software package version 14 (SAS institute Inc., Cary, NC). *p* < 0.05 (two-sided) was considered statistically significant.

## Results

### Clinicodemographic participant characteristics

Of the 105 participants, four withdrew because of physical condition and one changed the chemotherapy regimen after providing informed consent. Thus, 100 participants were included in the analysis. Table 1 shows the patients’ clinicodemographic characteristics. The mean age was 53.9 years (SD = 13.1), 79% of participants were women with breast cancer, 20% had had chemotherapy previously, and 80% were nonsmokers. The most common taxane used was PTX (60%: for breast cancer, administered weekly), followed by nab-PTX (21%: for pancreatic cancer, administered weekly for 3 weeks with 1 week off, or every other week) and DTX (19%: for breast cancer, administered every 3 weeks).

**Table 1:** Patient characteristics

Characteristics	Total		Without taste alteration		With taste alteration		*p*-value
	(*n*=100)		(*n*=41)		(*n*=59)		
	*n* (%)		*n* (%)		*n* (%)		
Age							
Mean (SD)	53.9	(13.1)	55.2	(12.8)	52.9	(13.4)	0.36^c^
Gender							
Female	80	(80.0)	31	(75.6)	49	(83.1)	0.36^b^
Male	20	(20.0)	10	(24.4)	10	(17.0)	
Type of cancer							
Breast	79	(79.0)	31	(75.6)	48	(81.4)	0.49^b^
Pancreas	21	(21.0)	10	(24.4)	11	(18.6)	
Stage							
1	8	(8.0)	6	(14.6)	2	(3.4)	0.06^a^
2	33	(33.0)	9	(22.0)	24	(40.7)	
3	12	(12.0)	4	(9.8)	8	(13.6)	
4	47	(47.0)	22	(53.7)	25	(42.4)	
Purpose of therapy							
Neo-adjuvant	36	(36.0)	15	(36.6)	21	(35.6)	0.15^a^
Adjuvant	20	(20.0)	5	(12.2)	15	(25.4)	
Recurrence	22	(22.0)	8	(19.5)	14	(23.7)	
Diagnostic stage 4	22	(22.0)	13	(31.7)	9	(15.3)	
Chemotherapy agent							
PTX*	60	(60.0)	30	(73.2)	30	(50.8)	0.002^a^
DTX^†^	19	(19.0)	1	(2.4)	18	(30.5)	
nab-PTX^‡^	21	(21.0)	10	(24.4)	11	(18.6)	
Previous chemotherapy							
No	80	(80.0)	39	(95.1)	41	(69.5)	0.0016^a^
Yes	20	(20.0)	2	(4.9)	18	(30.5)	
Median surface area (m^2^, 25–75%)							
PTX	1.5	[1.4–1.6]	1.5	[1.4–1.6]	1.5	[1.4–1.6]	0.90^c^
DTX	1.6	[1.4–1.7]	1.3	[1.3–1.3]	1.6	[1.4–1.7]	0.17^c^
nab-PTX	1.6	[1.5–1.7]	1.6	[1.5–1.7]	1.7	[1.6–1.7]	0.25^c^
Median number of treatment (25–75%)							
PTX	10	[8–12]	10	[8–11.8]	10.5	[8.8–12.3]	0.43^c^
DTX	3	[2–3]	3	[3–3]	3	[2–3.3]	0.85^c^
nab-PTX	10	[7.5–17]	10.5	[7–22.5]	10	[8–12]	0.85^c^
Median total dosage (mg, 25–75%)							
PTX	1200	[1000–1424]	1200	[880–1451]	1225	[1063–1446]	0.49^c^
DTX	330	[252–381]	309	[309–309]	333	[249–382]	0.78^c^
nab-PTX	1890	[1356–3200]	1945	[1311–4743]	1730	[1352–2200]	0.42^c^
Living situation							
Living alone	10	(10.0)	6	(14.6)	4	(6.8)	0.31^a^
Living with someone	90	(90.0)	35	(85.4)	55	(93.2)	
Occupational status							
Working	49	(49.0)	20	(48.8)	29	(49.2)	0.97^b^
Not working	35	(35.0)	14	(34.1)	21	(35.6)	
Sick leave	16	(16.0)	7	(17.1)	9	(15.3)	
Smoking habit							
None smoker	80	(80.0)	33	(80.5)	47	(79.7)	1.00^a^
Quitting temporary	10	(10.0)	4	(9.8)	6	(10.2)	
Smoking	10	(10.0)	4	(9.8)	6	(10.2)	
Cooking responsibility							
The participant	59	(59.0)	24	(58.5)	35	(59.3)	0.99^b^
Shared responsibility	19	(19.0)	8	(19.5)	11	(18.6)	
Rarely cook	22	(22.0)	9	(22.0)	13	(22.0)	

*SD* standard deviation, *DTX* docetaxel, *nab-PTX* nab-paclitaxel, *PTX* paclitaxel

^a^Fisher’s exact test

^b^Chi-squared test

^c^Mann-Whitney *U*-test

^d^Student’s *t*-test

*Monotherapy for 41 participants, with molecular targeted agent for 19 participants, administered weekly (1 cycle = 3 administrations)

^†^Monotherapy for 10 participants, with cyclophosphamide for 4 participants, with molecular targeted agent for 5 participants, administered every 3 weeks (1cycle = 1 administration)

^‡^With gemcitabine for 21 participants, administered weekly for 3 weeks, with 1 week off, or every other week (1cycle =3 administrations)

### Association with the characteristics and presence of TAs

Among the 100 participants, 59% (*n* = 59) reported TAs, while 41% (*n* = 41) did not (Table 1). Treatment with chemotherapeutic agents and a previous chemotherapy were significantly associated with the presence of TAs (*p* = 0.002 and *p* = 0.0016, respectively). Our participants had various stages and purpose of therapy and, therefore, the number of treatments received prior to data collection varied (minimum to maximum times): 6 to 113 with PTX, 2 to 19 with DTX, and 6 to 62 with nab-PTX (data not shown). However, the stage, purpose of therapy, and the median number of treatments were not associated with the presence of TAs. Moreover, age and smoking habit were not associated with the presence of TAs.

### Characteristics of TAs

The prevalence of TAs was the highest in patients receiving DTX (18/19; 95%), followed by that in patients receiving nab-PTX (11/21; 52%) and PTX (30/60; 50%) (Table 1). Table 2 shows the severity and duration of the TAs. Regarding severity, approximately 90% reported mild or moderate TAs with PTX and nab-PTX therapy. For DTX therapy, approximately 70% reported moderate or severe TAs. Approximately 40% of participants reported TAs lasting for 1–3 days with PTX or nab-PTX therapy, and 50% reported TAs lasting for >8 days with DTX therapy.

**Table 2:** Severity and duration of taste alterations by taxane agents

	Mild	Moderate	Severe	Very sever		1~3 days	4~7 days	8~14 days	Over 15 days
PTX (*n*=30)	17 (57%)	9 (30%)	3 (10%)	1 (3%)	PTX (*n*=29)*	11 (38%)	18 (62%)	0 (0%)	0 (0%)
DTX (*n*=18)	4 (22%)	8 (44%)	5 (28%)	1 (6%)	DTX (*n*=18)	2 (11%)	7 (39%)	5 (28%)	4 (22%)
nab-PTX (*n*=11)	9 (82%)	1 (9%)	0 (0%)	1 (9%)	nab-PTX (*n*=11)	5 (45%)	4 (36%)	2 (18%)	0 (0%)

*PTX* paclitaxel, *DTX* docetaxel, *nab-PTX* nab-paclitaxel

*Missing data for 1 participants

### Objective TA findings

Table 3 shows the association between TRTs and TAs. Overall, the TRTs of all tastants in the non-TA group were <3.5 (normal). In the TA group, only the TRTs of salty and bitter tastants were ≥3.5 (hypogeusia) and were significantly higher than those in the non-TA group (3.8 vs 3.2, *p* = 0.045; 3.5 vs 3.0, *p* = 0.049, respectively).

**Table 3:** Means and SDs of TRT* in participants with and without taste alterations by taxane agents

	Total (*n*=100)			PTX (*n*=41)			DTX^†^ (*n*=19)			nab-PTX (*n*=21)		
	TA (−)	TA (+)	*p*-value	TA (−)	TA (+)	*p*-value	TA (−)	TA (+)	*p*-value	TA (−)	TA (+)	*p*-value
	(*n*=41)	(*n*=59)		(*n*=30)	(*n*=30)		(*n*=1)	(*n*=18)		(*n*=10)	(*n*=11)	
	Mean (SD)			Mean (SD)			Mean (SD)			Mean (SD)		
Sweet	3.2 (1.2)	3.5 (1.1)	0.10	3.1 (1.2)	3.2 (1.1)	0.49	2.5 (-)	3.2 (0.9)	NA	3.4 (1.3)	4.7 (1.0)	0.022
Salty	3.2 (1.5)	3.8 (1.3)	0.045	3.2 (1.5)	3.5 (1.1)	0.35	2.0 (-)	3.7 (1.4)	NA	3.2 (1.5)	4.5 (1.5)	0.063
Sour	2.8 (0.9)	3.1 (1.1)	0.31	2.8 (1.0)	2.8 (1.0)	0.82	2.5 (-)	3.0 (0.8)	NA	2.9 (0.8)	4.0 (1.2)	0.020
Bitter	3.0 (1.1)	3.5 (1.2)	0.049	3.0 (1.0)	3.1 (1.1)	0.77	2.5 (-)	3.5 (1.0)	NA	3.1 (1.5)	4.5 (1.4)	0.039

*SD* standard deviation, *TRT* taste recognition thresholds, *PTX* paclitaxel, *DTX* docetaxel, *nab-PTX* nab-paclitaxel, *TA* taste alteration

*Two measurement points; the chorda tympani nerve field which is branch of the facial nerve (2cm left edge from the tip of the tongue), the glossopharyngeal nerve field (near the circumvallate papillae)

^†^DTX could not be statistically interpreted because only one participant reported TAs

In all three chemotherapeutic agents, the TRTs of all tastants in the non-TA group were also <3.5 (normal). In the TA group, the tastants had TRTs of ≥3.5 (hypogeusia), as follows: salty (3.5) in PTX; salty (3.7) and bitter (3.5) in DTX; and sweet (4.7), salty (4.5), sour (4.0), and bitter (4.5) in nab-PTX. Only sweet, sour, and bitter tastants in nab-PTX had significantly higher TRTs than those in the non-TA group (4.7 vs 3.4, *p* = 0.022; 4.0 vs 2.9, *p* = 0.020; 4.5 vs 3.1, *p* = 0.039, respectively). The other TRTs in the TA group were within the normal range.

### Subjective TA findings

Table 4 shows the association between the CiTAS dimension score and TAs. Overall, the mean scores of the four dimensions in the non-TA group were approximately 1.0 (normal taste). The scores were significantly higher in the TA group than those in the non-TA group. In all three chemotherapeutic agents, the CiTAS dimension scores in the non-TA group were also approximately 1.0. The scores were higher in the TA group than in the non-TA group. PTX- and nab-PTX-treated patients showed significantly higher scores in all dimensions except for discomfort in nab-PTX-treated patients.

**Table 4:** Means and SDs of CiTAS four dimensions scores in participants with and without taste alterations by taxane agents

	Total (*n*=100)			PTX (*n*=41)			DTX* (*n*=19)			nab-PTX (*n*=21)		
	TA (−)	TA (+)	*p*-value	TA (−)	TA (+)	*p*-value	TA (−)	TA (+)	*p*-value	TA (−)	TA (+)	*p*-value
	(*n*=41)	(*n*=59)		(*n*=30)	(*n*=30)		(*n*=1)	(*n*=18)		(*n*=10)	(*n*=11)	
	Mean (SD)			Mean (SD)			Mean (SD)			Mean (SD)		
Decline in basic taste	1.00 (0)	1.96 (0.91)	<0.001	1.00 (0)	1.81 (0.77)	<0.001	1.00 (-)	2.32 (0.96)	NA	1.00 (0)	1.75 (1.10)	<0.001
General taste alterations	1.02 (0.12)	2.04 (0.85)	<0.001	1.03 (0.14)	1.93 (0.78)	<0.001	1.00 (-)	2.34 (0.80)	NA	1.00 (0)	1.86 (1.03)	<0.001
Phanto-and parageusia	1.00 (0)	1.70 (0.80)	<0.001	1.00 (0)	1.80 (0.87)	<0.001	1.00 (-)	1.79 (0.77)	NA	1.00 (0)	1.30 (0.55)	0.045
Discomfort	1.20 (0.35)	1.50 (0.58)	0.001	1.20 (0.40)	1.41 (0.42)	0.006	1.17 (-)	1.67 (0.79)	NA	1.20 (0.17)	1.45 (0.52)	0.37

*PTX* paclitaxel, *DTX* docetaxel, *nab-PTX* nab-paclitaxel, *TA* taste alteration, *SD* standard deviation

*DTX could not be statistically interpreted because only one participant reported TAs

In the TA group, the most commonly affected dimension was general TAs (PTX, 1.93; DTX, 2.34; nab-PTX, 1.86), followed by decline in basic taste (PTX, 1.81; DTX, 2.32; nab-PTX, 1.75). The most commonly affected items in the “general TAs” dimension were “Have difficulty tasting food” (PTX, 2.21; DTX, 3.00; nab-PTX, 2.27) and “Food doesn’t taste as it should” (PTX, 2.23; DTX, 2.59; nab-PTX, 1.82) (data not shown). Similarly, “Have difficulty tasting umami ” (PTX, 2.27; DTX, 2.67; nab-PTX, 2.09) and “Have difficulty tasting salty ” (PTX, 2.00; DTX, 2.67; nab-PTX, 1.82) were the most commonly affected items in the “decline in basic taste” dimension (data not shown). Moreover, smell or flavor perception was significantly higher in the TA group (PTX, 1.57 vs 1.10, *p* = 0.002; DTX, 1.89 vs 1.00, *p* = NA; nab-PTX, 1.82 vs 1.00, *p* = 0.008) (data not shown). The scores for DTX in the TA group tended to be higher than those for the other two agents.

### Association of TAs with appetite, weight change, QOL, K6 score, and adverse events

Table 5 shows the association of TAs with appetite, weight change, QOL, psychological distress, and adverse events. Overall, the scores for “appetite,” “mouth or throat sores,” and “dry mouth” were significantly higher in the TA group than those in the non-TA group (1.22 vs 0.41, *p* < 0.001; 0.59 vs 0.22, *p* = 0.016; 0.86 vs 0.35, *p* = 0.010, respectively).

**Table 5:** Means of appetite, weight change, QOL, K6, and adverse events in participants with and without taste alterations by taxane agents

	Total (*n*=100)			PTX (*n*=41)			DTX* (*n*=19)			nab-PTX (*n*=21)		
	TA (−)	TA (+)	*p*-value	TA (−)	TA (+)	*p*-value	TA (−)	TA (+)	*p*-value	TA (−)	TA (+)	*p*-value
	(*n*=41)	(*n*=59)		(*n*=30)	(*n*=30)		(*n*=1)	(*n*=18)		(*n*=10)	(*n*=11)	
Appetite	0.41	1.22	<0.001	0.37	0.97	0.007	1.00	1.44	NA	0.50	1.55	0.10
Weight change	1.47	0.29	0.35	0.50	-0.14	0.49	-2.70	0.50	NA	4.80	1.00	0.010
FACT-G total	74.00	70.7	0.22	76.43	69.86	0.077	58.00	74.77	NA	68.30	66.27	0.60
Physical	3.05	2.76	0.063	3.15	2.65	0.01	1.86	2.97	NA	2.84	2.70	0.78
Social	2.74	2.67	0.43	2.81	2.74	0.51	3.50	2.81	NA	2.45	2.24	0.38
Emotional	2.95	2.90	0.69	2.98	2.89	0.75	1.00	3.06	NA	3.05	2.68	0.21
Functional	2.65	2.57	0.55	2.80	2.51	0.19	2.57	2.67	NA	2.20	2.55	0.38
K6	4.46	4.81	0.46	4.40	4.80	0.64	11.00	4.39	NA	3.33	3.64	0.41
Nausea	0.24	0.44	0.13	0.27	0.47	0.24	0.00	0.61	NA	0.20	0.09	0.94
Mouth or throat sores	0.22	0.59	0.016	0.23	0.37	0.27	0.00	1.06	NA	0.20	0.45	0.40
Dry mouth	0.35	0.86	0.010	0.37	0.57	0.25	0.00	1.11	NA	0.33	1.27	0.041
Numbness or tingling in hands or feet	1.17	1.57	0.11	1.07	1.73	0.019	0.00	1.06	NA	1.60	2.00	0.39

*PTX* paclitaxel, *DTX* docetaxel, *nab-PTX* nab-paclitaxel, *TA* taste alteration, *SD* standard deviation, *FACT-G* Functional Assessment of Cancer Therapy-General

*DTX could not be statistically interpreted because only one participant reported TAs

As with PTX, the scores for “appetite” (0.97 vs 0.37, *p* = 0.007) and “numbness or tingling in the hands or feet” (1.73 vs 1.07, *p* = 0.019) were significantly higher, and the score for the “physical dimension” in the FACT-G was significantly lower (2.65 vs 3.15, *p* = 0.01) in the TA group than those in the non-TA group. Regarding nab-PTX, the score for “dry mouth” was significantly higher in the TA group than in the non-TA group (1.27 vs 0.33, *p* = 0.041). There was no statistical relationship between TAs and other variables.

With regard to the objective TAs with appetite, weight change, QOL, psychological distress, and adverse events, we did not find any association other than the following: low correlation between salt and dry mouth (*ρ* = 0.25, *p* = 0.01, the greater the decrease in sensitivity to salt, the stronger was the dry mouth feeling), low correlation between sour and nausea (*ρ* = −0.22, *p* = 0.03, the greater the sensitivity to sourness, the stronger the nausea).

### Association between TA characteristics and appetite

There were low to moderate correlations between decreased appetite and the following CiTAS items: “Food doesn’t taste as it should” (*ρ* = 0.49, *p* < 0.001), “Everything tastes bad” (*ρ* = 0.38, *p* < 0.001), and “Have difficulty tasting food” (*ρ* = 0.32, p = 0.001), all of which belong to the “general TAs” dimension, and “Have difficulty tasting umami ” (*ρ* = 0.38, *p* < 0.001), which belongs to the “decline in basic taste” dimension (data not shown).

### Predictors of TAs

To identify the putative predictors of TAs, univariate and multivariate logistic regression analyses were performed (Table 6). Age and sex were included as basic explanatory variables and as potential predictors. The analyses revealed that the type of agent (DTX vs PTX: adjusted odds ratio [aOR], 11.26; 95% confidence interval [CI], 1.50–238.36; *p* = 0.015; DTX vs nab-PTX: aOR, 23.50; 95% CI, 2.55–570.10; *p* = 0.004), previous chemotherapy (aOR, 5.44; 95% CI, 1.02–44.20; *p* = 0.047), dry mouth (aOR, 2.58; 95% CI, 1.31–5.85; *p* = 0.005), and numbness or tingling in the hands or feet (aOR, 2.04; 95% CI, 1.25–3.57; *p* = 0.004) were significantly associated with TAs.

**Table 6:** Multiple logistic regression analysis of possible predictors for taste alterations

Predictor	Univariate analysis				Multivariate analysis			
	OR	95%Cl		*p*-value	Adjusted OR	95%Cl		*p*-value
Female vs male	1.58	0.59	4.23	0.36	-	-	-	-
Age	0.99	0.96	1.02	0.39	-	-	-	-
Type of cancer (breast vs pancreas)	1.41	0.53	3.71	0.49	-	-	-	-
Stage (3 or 4 vs 1 or 2)	0.61	0.27	1.36	0.23	-	-	-	-
Surface area	3.64	0.21	73.16	0.38	-	-	-	-
Number of treatment	0.99	0.96	1.02	0.44	-	-	-	-
Total dosage	1.00	1.00	1.00	0.12	-	-	-	-
DTX vs PTX	18.00	2.26	143.56	0.006	11.26	1.50	238.36	0.015
DTX vs nab-PTX	16.36	1.83	145.95	0.012	23.50	2.55	570.10	0.004
Previous chemotherapy (yes vs no)	8.56	1.86	39.35	0.006	5.44	1.02	44.20	0.047
Nausea	1.49	0.84	2.93	0.18	-	-	-	-
Dry mouth	2.11	1.17	3.79	0.004	2.58	1.31	5.85	0.005
Mouth or throat sores	2.23	1.13	4.34	0.01	-	-	-	-
Numbness or tingling in hands or feet	1.43	0.96	2.12	0.068	2.04	1.25	3.57	0.004
Living situation (living with someone vs living alone )	2.36	0.63	9.78	0.20	-	-	-	-
Occupational status (working vs sick leave or not working )	1.02	0.46	2.26	0.97	-	-	-	-
Smoking habit (smoking vs quitting temporary or not smoking)	1.05	0.28	4.34	0.95	-	-	-	-
Cooking responsibility (the participant or shared responsibility vs rarely cook)	1.00	0.37	2.59	0.99	-	-	-	-

AUC: 0.81, *PTX* paclitaxel, *DTX* docetaxel, *nab-PTX* nab-paclitaxel, *OR* odds ratio, *95%Cl* 95% confidence interval

## Discussion

In this study, the prevalence of TAs associated with taxane-based chemotherapies was 59%, with DTX being associated with a higher prevalence of and more severe and longer TAs. These findings are consistent with previous results [21, 31, 32], suggesting that DTX regimens are more likely to cause TAs than PTX or nab-PTX regimens.

In the objective evaluations of nab-PTX-treated patients, significantly elevated TRTs (hypogeusia) were observed only in the TA group. This result might be associated with the occurrence of dry mouth in the TA group receiving nab-PTX. Dry mouth is caused by low saliva flow from the salivary glands [15]. Previous studies have shown an association between TAs and dry mouth [7, 21, 33]. Hyposalivation may reduce the intensity of taste sensations due to a limited ability to dissolve food particles, thereby reducing the number of molecules reaching the taste receptors [34] and taste perception might be reduced. In addition, patients receiving nab-PTX are only with pancreatic cancer that is probably higher staged of the disease due to clinical indication of nab-PTX for pancreatic cancer. Taste alteration itself can be a symptom of the disease in such advanced stage [35]. Regarding DTX, the timing of objective evaluation was likely too late to capture the actual TAs because chemotherapeutic disturbance toward the turn over cycle of taste receptor cells seem to recover 16–21 days post-injection [36]. Regarding PTX, it remains unknown why the TRTs in the TA group were almost in the normal range. The fact that the data collection time point in this study varied, such as 7 (for PTX/nab-PTX), 14 (nab-PTX), or 21 days (DTX), after administration made it especially difficult to interpret the findings from the objective taste evaluation. Previous studies of breast cancer patients undergoing anthracycline and/or taxane-containing adjuvant chemotherapy have shown that objective TAs were largest 4–6 days after chemotherapy administration [6]. In our study, therefore, the objective taste evaluation at 14 and 21 days after administration might not reflect the real TAs. In future studies, objective taste assessments should be conducted early after treatment administration, and the participants’ data collection time points should be the standardized.

In the subjective evaluation (CiTAS), for all three agents, the TA group reported more severe taste-related symptoms than did the non-TA group. Such discrepancy between objective and subjective evaluations of TAs was also observed in previous studies [33, 37]. Subjective TA findings do not always correspond to findings by objective measures, which examine the function of taste per se, because people mostly refer to the overall flavor of food (combination of taste, smell, texture, and temperature) when asked about its taste [37]. In our participants, the subjective smell or flavor perception in the TA group deteriorated; therefore, obvious TAs might be reported more often with subjective evaluation.

In this study, five items of the CiTAS were associated with decreased appetite. A previous study showed that specific subjective TAs are related to decreased appetite and decreased calorie and nutrient intake [5]. Future studies on the relationship between specific subjective TAs and appetite and nutritional issues are therefore needed to elucidate these findings.

We found no association between TAs and weight change. Previous studies have demonstrated conflicting findings regarding the association between chemotherapy-related TAs and weight change. While some studies showed no differences [38–40], others showed decreased weight associated with specific TAs [5, 10]. In our study, most of the TAs were not severe. Moreover, the TAs were temporary and were mitigated at the end of the chemotherapy cycle; thus, participants might have maintained their oral intake and weight.

We also did not find any association of TAs with psychological distress or QOL, except for the physical dimension in patients receiving PTX. The degree of distress or the impact on QOL related to TAs can vary individually. Some patients simply accept TAs as temporarily annoying symptoms, while others consider them as bothersome and distressing [4, 13, 32, 41]. The lack of association between TAs and QOL in our study might be due to the QOL scale used, which is designed to evaluate QOL in a recall period of 7 days. Some participants whose treatment schedule was every 14 days for nab-PTX or every 21 days for DTX underwent the QOL evaluation during the washout period; therefore, the reported results might have been underestimated. Furthermore, despite the validity of the QOL and psychological distress tools that we used, these might be inadequate to assess the various types of distress associated with TAs, such as psychosocial influence, loss of eating pleasure, or change in eating behaviors.

In this study, DTX therapy, previous chemotherapy, dry mouth, and numbness or tingling in the hands or feet were independently associated with TAs. However, the larger CIs for DTX therapy and previous chemotherapy reveal an uncertainty of the estimates. Damage to peripheral nerve is one of the mechanisms of TAs [14]. Although our findings imply that individuals who report a “glove and stocking” type of neuropathy are more likely to experience TAs, this does not mean actual damage to peripheral nerves that convey taste perception (facial, glossopharyngeal, or vagus nerve). Hence, further research is needed to confirm the association between TAs and neuropathy.

This study has some limitations. First, a causal relationship between chemotherapy and TAs could not be established because of the absence of baseline taste evaluation. Besides, the long-term effects of TAs and the association of appetite, weight changes, and QOL are unknown because our study was a cross-sectional study. Second, there could exist confounding factors between PTX or DTX and nab-PTX due to disease characteristics of breast and pancreatic cancers, as well as combined agents such as gemcitabine, trastuzumab, pertuzumab, bevacizumab, and steroids. Breast and pancreatic cancers have very different clinical courses and symptoms. In addition, the number of treatments that were administered at the time of data collection in participants varied widely, which implies that participants were in various chemotherapy trajectories. These heterogeneities could be confounding factors for the study endpoints, especially with regard to adverse events, weight change, and functional status. Third, statistical analysis was not possible for DTX because only one participant reported not having TAs. Fourth, the umami taste was not included in the objective evaluation because no validated measuring tool has been established for it. Umami is a tastant that indicates the protein content [37] and may have a significant impact on diet, appetite, and weight. Lastly, we did not collect information on pungent and spicy perceptions, which are qualitative tastes that are potentially impacted by neuropathy, because we focused on the CiTAS evaluations for subjective TAs; besides, there is no validated objective and subjective evaluation tool for pungent and spicy perceptions. Moreover, we did not measure the saliva volume in consideration of the physical burden of the participants, and did not consider the collection of information on oral hygiene and bad breath. We used only the PRO-CTCAE for the evaluation of adverse events and, therefore, mucositis (which is “mouth or throat sores” in PRO-CTCAE) was not assessed using a specific scale. Those variables might be critical influential factors of TAs. We recommend further prospective studies exploring the exact mechanism underlying TAs and using both objective and subjective methods with appropriate evaluation timing and variables. The association of specific types of TAs with appetite, dietary behavior, nutrition, and QOL across various cancer types and chemotherapy regimens is also worthy of further investigation. Propensity score may be one of the statistical methods used to adjust covariates in observational studies that are difficult to randomize and prone to various confounding.

In conclusion, approximately 60% of participants treated with taxane-based regimens reported TAs, with DTX associated with the highest prevalence of TAs. TAs affected the patient’s appetite, not their weight or QOL. We found that DTX therapy, previous chemotherapy, dry mouth, and peripheral neuropathy were independent risk factors for TAs. Due to the homogeneity in timing and nature of the chemotherapy received by the study participants, this study only reinforces what is already conferred in the literature. Healthcare professionals play an important role in helping patients deal with chemotherapy-related adverse events. Through adequate counseling to patients receiving chemotherapy, healthcare professionals can provide specific information, such as the prevalence, timing of onset and duration, possible nature of alteration, and impact on appetite of TAs, in advance from the results of taste studies in people receiving chemotherapy; this can support well-equipped management strategies. An intervention study on the effects of counseling of taste alterations and coping strategies by healthcare professionals may be worth conducting in the future.

## Data Availability

The datasets of the current study are available from the corresponding author on reasonable request.

## Code availability

N/A
